# Exercise Induced Laryngeal Obstruction in Humans and Equines. A Comparative Review

**DOI:** 10.3389/fphys.2019.01333

**Published:** 2019-10-30

**Authors:** Zoe Louise Fretheim-Kelly, Thomas Halvorsen, Hege Clemm, Ola Roksund, John-Helge Heimdal, Maria Vollsæter, Constanze Fintl, Eric Strand

**Affiliations:** ^1^Faculty of Veterinary Medicine, Norwegian University of Life Sciences, Oslo, Norway; ^2^Department of Clinical Science, Faculty of Medicine, University of Bergen, Bergen, Norway; ^3^Department of Sports Medicine, Norwegian School of Sport Sciences, Oslo, Norway; ^4^Department of Pediatrics, Haukeland University Hospital, Bergen, Norway; ^5^Faculty of Health and Social Sciences, Western Norway University of Applied Sciences, Bergen, Norway; ^6^Department of Oral Surgery, Haukeland University Hospital, Bergen, Norway; ^7^Department of Clinical Science, University of Bergen, Bergen, Norway

**Keywords:** comparative medicine, Exercise Induced Laryngeal Obstruction (EILO), exercise laryngoscopy, exercise dyspnoea, larynx, equine upper airway disorders, dynamic laryngeal collapse

## Abstract

Dynamic obstructions of the larynx are a set of disorders that occur during exercise in equines and humans. There are a number of similarities in presentation, diagnosis, pathophysiology and treatment. Both equines and humans present with exercise intolerance secondary to dyspnea. During laryngoscopy at rest, the larynx appears to function normally. Abnormalities are only revealed during laryngoscopy at exercise, seemingly triggered by increased ventilatory demands, and quickly resolve after cessation of exercise. Lower airway disease (asthma being the most prevalent condition), cardiac disease and lack of fitness are the major differentials in both species. Laryngoscopic examination during exercise should be performed from rest to peak exertion to allow for a comprehensive diagnosis, including where the airway collapse begins, and thereafter how it progresses. Dynamic disorders with most visual similarity between humans and equines are: aryepiglottic fold collapse (both species); equine dynamic laryngeal collapse (DLC) relative to some forms of human combined supraglottic/glottic collapse; and epiglottic retroversion (both species). Quantitative grading techniques, such as airway pressure measurement, that have proven effective in veterinary research are currently being piloted in human studies. Conditions that appear visually similar are treated in comparable ways. The similarities of anatomy and certain types of dynamic collapse would suggest that the equine larynx provides a good model for human upper respiratory tract obstruction during exercise. Thus, close collaboration between veterinarians and medical personal may lead to further advancements in understanding pathophysiologic processes, and enhance the development of improved diagnostic tests and treatments that will benefit both species.

## Dynamic Obstructions of the Upper Respiratory Tract in Humans and Equines

Human medicine recently agreed upon the phrase Exercise Induced Laryngeal Obstruction (EILO) (Christensen et al., 2015; Halvorsen et al., 2017) to describe the phenomenon that in equines is usually referred to as dynamic obstruction of the upper respiratory tract (Lane et al., 2006a). These conditions represent comparable sets of disorders characterized by the larynx appearing normal at rest, with abnormalities seemingly induced by the increased ventilatory demands during ongoing exercise, and thereafter quickly resolving with cessation of exercise.

## Comparative Medicine

*Between animal and human and medicine there is no dividing line – nor should there be. The object is different but the experience obtained constitutes the basis of all medicine*, Rudolf Virchow (1821–1902). Comparative medicine is the study of comparable diseases in different species; the similarities give the model its relevance, but the differences often provide the most informative insights. By studying pathologies that *co-exist* in humans and animals, we may uncover common denominators of disease and identify new therapies, while at the same time reducing animal welfare concerns. Disease is not inflicted on these animals; instead they may in fact benefit from early access to new diagnostics, treatments and preventive techniques.

## Why Involve Equines in the Study of EILO in Humans?

Equines and humans are the predominant species that compete at maximal exertion and additionally have documented laryngeal abnormalities associated with exercising at high intensity. As such, their upper respiratory tracts are subject to similar strong aerodynamic stresses, potentially also providing a common platform for research and understanding. Dynamic upper respiratory tract obstructions and their treatment are far better described in equines than in humans, suggesting a potential for added benefit for humans. This article compares current knowledge on presentation, diagnostics and treatment of this phenomenon, aiming to uncover common features that might lead to better treatments in both species.

## Search Strategy

Guidelines set out by Preferred Reporting Items for Systematic Review and Meta-Analyses (PRISMA) where used for guidance. The Cochrane Database, Ovid Medline, PubMed, and Web of Science where searched using three search domains for equine disease and four domains for human disease. Each search domain was combined using “AND” and within each domain combined with “OR.” The first domain for equine disease included, “Dynamic,” “maximal exercise,” “treadmill exercise,” and “poor performance.” Second domain, “Upper respiratory tract,” “soft palate,” “DDSP,” “epiglottis,” “laryngeal,” “aryepiglottic fold,” “dynamic laryngeal collapse (DLC),” “arytenoid,” “upper airway,” and “vocal fold.” Third domain “collapse,” “disease,” “obstruction,” “stridor,” and “respiratory noise.” For human, “Exercise,” “exertional,” “maximal exercise,” “exercised-induced,” “athletes,” “episodic,” “treadmill,” “high-intensity exercise,” “paradoxical,” and “functional.” Second domain, “induced,” “inducible,” “stimulated,” and “provoked.” Third domain, “laryngeal,” “vocal cord,” “vocal fold,” “larynx,” “glottis,” “upper respiratory tract,” “laryngomalacia,” “soft palate,” and “upper airway.” Fourth domain, “obstruction,” “dysfunction,” “dyspnoea,” “stridor,” “obstruction,” “inspiratory,” “laryngeal dyskinesis,” “asphyxia,” “upper airway obstruction,” and “vocal fold dysfunction.” Last search date was 27/12/2018. Reference lists from included studies were checked for other studies and Ph.D. thesis on dynamic collapse from NMBU regarding equines and Bergen University/Haukeland University Hospital regarding humans were used. The search results were first scanned by title and inappropriate articles removed. Abstracts of remaining articles were read for relevance, and those articles most appropriate to poor performance and dyspnea due to laryngeal obstruction at exercise in equines and humans were read fully. The information obtained from reading these articles was then used to collate a comparative review of laryngeal induced dyspnea at exercise in equines and humans.

## Clinical Presentation

In both equines and humans, the common presenting complaint is reduced exercise tolerance occurring secondary to dyspnea, which is typically characterized by inspiratory stridor that worsens as exercise intensity increases and resolves within a few minutes of cessation of exercise (Newman et al., 1995; Beard, 1996; Rundell and Spiering, 2003; Lane et al., 2006a). In humans, panic reactions occasionally occur as a response to breathlessness (Roksund et al., 2009). Anecdotally, corresponding “stress and avoidance behaviors” have been reported in equines, associated with occasions of dyspnea at racetracks.

In humans, first time presentation of EILO is most frequent in active youngsters, with those partaking in competitive sport being overrepresented (Roksund et al., 2009; Nielsen et al., 2013). Symptoms might initially be vaguely described; however, on closer questioning some very typical features will be revealed. The inspiratory phase of the breathing cycle being more affected than the expiratory phase, and symptoms are at their worst when ventilation requirements are at their most intense, with symptoms typically abating as ventilation decreases – unless panic occurs. This pattern clearly contrasts the expiratory dyspnea of Exercise Induced Bronchoconstriction (EIB) that peaks after cessation of exercise. Nevertheless, these conditions are too frequently confused, often with unfortunate consequences (Chiang et al., 2008; Roksund et al., 2017). However, care must be taken when interpreting self-reported symptoms, as many patients find it hard to attribute symptoms to a particular phase of respiration, and a number of studies suggest that self-reported symptoms are a poor prediction of EIB and EILO (Nielsen et al., 2013; Johansson et al., 2014).

Similarly, symptoms of dynamic obstruction of the upper respiratory tract in equines often present around 2–3 years of age, as performance expectations increase (Lane et al., 2006a). Trainers report the horse not exerting itself as fully as before, abnormal respiratory noise at higher exercise intensities, and poorer race performance in terms of placings and earnings (Beard, 1996). Again, upon closer questioning, trainers and jockeys can often time the respiratory noise to inspiration (Lane et al., 2006a). Abnormal breathing patterns may occur, with uncoupling of the typical locomotor respiratory coupling mechanism (LRC), which in galloping horses is 1:1 (Fitzharris et al., 2015). Horses with respiratory tract disease have been shown to adopt a 2:1 pattern when galloping, taking 1 breath over 2 strides (Fitzharris et al., 2015). This may reflect “breathlessness” and an effort to regulate pathological dyspnea. By increasing the time over which a breath is inhaled, flow rate and pressures within the airway can be reduced, thereby exerting less stress on the upper airway structures (Fitzharris et al., 2015). No such adaptations have been reported in humans with EILO, although change in timing of the respiratory cycle is an area of research interest in this patient group.

## Diagnosis

Dynamic obstructions by definition require the upper airways to appear normal at rest, and that abnormal function is revealed during ongoing exercise. Thus, visualization of the larynx *during* exercise is required for a diagnosis in both species, allowing the clinicians to determine which structures are implicated, as well as when and in what sequence they become involved (Morris and Seeherman, 1991; Lane et al., 2006b; Roksund et al., 2017).

In equines treadmill endoscopy protocols have been in use for more than three decades (Morris and Seeherman, 1991). All apply the same principles of running the horse at the trot or gallop, until symptoms are seen or to fatigue, with an appropriately positioned endoscope in place during the entire test (Lane et al., 2006a). Most protocols include a method to determine the level of exertion, such as a heart rate monitor, ECG or gas flow analysis. Some centers include periods of free head carriage and periods of poll flexion to mimic real race or riding conditions, as recent articles report that head position can induce or aggravate many disorders (Strand et al., 2012). Pressure readings from the trachea and pharynx have become important tools at University clinics, to allow objective, precise measurement of the degree of inspiratory and/or expiratory airflow obstruction (Nielan et al., 1992; Ducharme et al., 1994). This has allowed the development of computational flow dynamics models, and consequently a better understanding of pathological processes and treatment planning (Rakesh et al., 2008a).

During the last decade overground endoscopes with GPS tracking have been developed, which allows the videoendoscopic test to be performed in the field (Franklin et al., 2008). Although overground endoscopy cannot be standardized as a treadmill test can, it does allow testing in the same environment in which symptoms occur. This allows evaluation of cases that are not suitable for, cannot come to the treadmill or do not get symptoms during treadmill testing.

The major differentials and co-morbidities for exertional dyspnea in the equine are lower airway problems, cardiac disease and poor fitness. Bronchoalveolar lavage is routinely performed as part of a respiratory performance workup to determine lower airway inflammatory disease status. ECG recordings during treadmill testing allow evaluation of any arrhythmias that may be present. It should be noted, however, that arrhythmias are commonly reported in racehorses and their clinical significance varies (Jose-Cunilleras et al., 2006; Allen et al., 2016).

In humans the continuous laryngoscopy exercise (CLE) test was similarly developed to study the visual presentation of the larynx during ongoing exercise in patients complaining of symptoms that on close questioning appeared to originate in the upper airways. The CLE test as described by Heimdal et al. (2006), is a complete incremental cardiopulmonary exercise test; including tidal flow volume loops, breath-by-breath gas analyses, ECG, video of the upper torso and head, and sound recordings, combined with an appropriately positioned endoscope in place for the entire test, with all data synced into one single file to allow storage and subsequent analyses (Heimdal et al., 2006). A number of modes of exercise can be used, but the CLE test is most often performed on a treadmill or an ergometer cycle controlled via software to produce a standardized exercise test. As with equine patients, replicating real life conditions where symptoms occur is useful and as such, laryngoscopy during rowing, stair climbing and even swimming have been described (Panchasara et al., 2015; Walsted et al., 2017). The benefit of this is the recreation of the conditions in which symptoms occur; the disadvantage is that these modes of exercise are harder to standardize, which poses a problem when evaluating effects from interventions or when conducting research.

As with equine patients, it is fundamental to view the larynx throughout the complete exercise test and recovery. This allows identification of the structures that are implicated, when and in what sequence they become involved and as such, a full visual representation on how the situation evolves; information that collectively is critical for appropriate treatment. For example, failing to observe excessive supraglottic tissue collapse prior to a glottic obstruction may result in patients receiving conservative treatment that may not be efficient, excessive supraglottic tissue obstruction is usually better treated by surgery (although the glottic component also needs to be addressed). Likewise in equines, failure to diagnose aryepiglottic fold collapse (MDAF) being secondary to failure of full arytenoid abduction and thus only treating the MDAF, will not result in full clinical improvement and return to performance. Thus, for both equines and humans, video laryngoscopy performed throughout an exercise test is by definition required to make a definitive diagnosis, and also imperative to institute appropriate treatment, although so far only in equines are these images supplemented by objective airway pressure measurements.

## Grading of Severity of Obstruction

**FIGURE 1:**
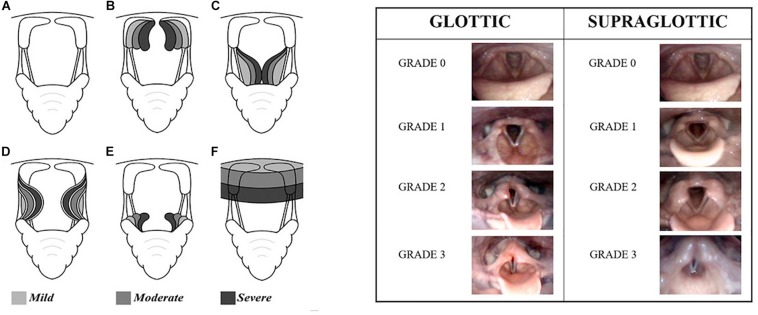
(Left) The equine grading system. Schematic illustration of **(A)** normal larynx, **(B)** arytenoid cartilage collapse, **(C)** vocal fold collapse, **(D)** medial deviation of aryepiglottic folds, **(E)** collapse of margins of epiglottis, and **(F)** pharyngeal roof collapse. Light gray: mild, gray: moderate, and dark gray: marked. With permission from K. Olstad. (Right) The human grading system according to Maat et al. (2009) illustrated by endoscopic photographic images during exercise from the larynx showing the different grades of laryngeal obstruction at the glottic and supraglottic levels.

Grading systems for the degree of obstruction caused by luminal collapse or medialization of the various structures in the upper airway (mainly the larynx), have been developed independently for both humans (Maat et al., 2009) and equines, and are surprisingly similar. These systems are to a large extent based on subjective visual assessments (Figure 1).

In equines, tracheal pressure measurements, first described by Nielan et al. (1992), have provided an objective measure for research for three decades (Nielan et al., 1992). Veterinary experiences with equine dynamic upper airway obstructions are currently guiding the development of better diagnostic tools for EILO in humans. The larynx functions as the entrance valve to the lower airway. Thus, techniques for tracheal and pharyngeal pressure measurements throughout a full CLE test in order to determine the normal and excessive pressure drops during high intensity exercise over this so vitally important valve, have been requested in a recent literature review (Halvorsen et al., 2017). Encouraged by the successes obtained by veterinarians in objectively quantifying obstructions in equines (Ducharme et al., 1994; Rehder et al., 1995; Holcombe et al., 1999), it is hoped that a better understanding of these pressure relationships will facilitate development of similar computer-based simulation models for humans as for horses. The first translaryngeal pressure measurements in humans during an exercise test have recently been performed, and proved to be feasible and tolerable as an objective outcome measure for obstruction of ventilation over the larynx (Fretheim-Kelly et al., 2019). Thus, application of equine veterinary research and practice has great potential to aid the understanding of the pathophysiology and treatment of EILO, and thereby contribute to the provision of quantifiable data that can be used to guide therapy.

## Pathophysiology Underlying Dynamic Upper Airway Collapse

The inciting cause of collapse varies and has not been determined for humans. In equines, a number of forms of dynamic collapse seem to be due to anatomic/functional phenotypes for which certain breeds seem predisposed (Woodie et al., 2005; Strand et al., 2012). However, all result in an initial local narrowing of the airway, which with further exercise often worsens. These pressure/flow/size relations are described mathematically by the Bernoulli principle and Hagen–Poiseuille’s formula. In simple terms, the narrower the lumen of a tube, the greater the velocity of the same volume of air compared with a tube of greater diameter, this in turn results in more negative transmural pressures. Thus, any structure that reduces the upper airway diameter will result in an increase in airflow velocity past this point, thereby increasing the lumen “collapsing pressures.” Susceptible structures are drawn into the airway, further reducing the lumen diameter and exacerbating the cycle (Rakesh et al., 2008b; Strand et al., 2009).

## Specific Pathologies

In humans, laryngeal obstruction is divided into supraglottic (structures rostral to the vocal folds) and glottic (at the level of the vocal folds) (Christensen et al., 2015). There is no such division in the equine. In the following paragraphs, each specific structure that is subject to collapse and thus potentially can obstruct the airflow, will be described and the treatment options discussed. Although each structure is described separately, most upper airway collapse involves multiple structures, this is termed “complex” airway collapse (Tan et al., 2005; Lane et al., 2006a; Strand and Skjerve, 2012).

## Supraglottic Collapse (Human) Versus Medial (Axial) Deviation of the Aryepiglottic Folds (MDAF) (Equine)

**FIGURE 2:**
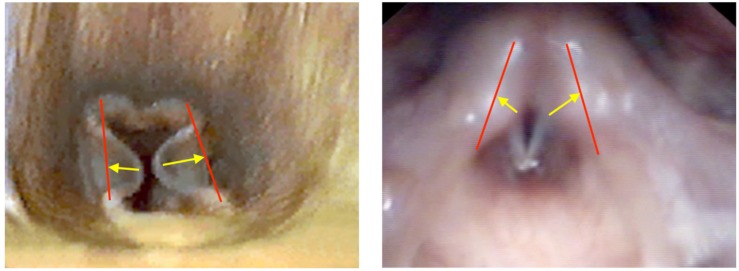
Endoscopic photographic images during exercise of: **(left)** marked medial deviation of the aryepiglottic folds in an equine. **(Right)** Grade 3 supraglottic EILO in a human. Red lines denote expected line of aryepiglottic fold in a normal subject. Yellow arrows denote degree of collapse to normal.

The aryepiglottic folds (AEF) are found both in humans and equines, and are membranous tissues that run from the epiglottis to the arytenoid cartilages, serving a protective function during swallowing (Negus, 1937). In both equines and humans they are supported by the corniculate cartilages and in humans and some equines by the cuneiform cartilages (Negus, 1937; King et al., 2001). In equines a number of mechanisms have been suggested to lead to MDAF; e.g., failure of full abduction of the arytenoids, lifting of the epiglottis, a structural weakness of the tissue, an excess of fold tissue or stretching of the fold tissue secondary to other upper airway obstructions, resulting in laxity (Franklin, 2008). The result is displacement of the folds axially (medially) during inspiration, as the lax tissue cannot withstand the increasingly negative pressure created by the increasing airflow induced by the increased ventilation of exercise (King et al., 2001). This is supported by research using computer modeling, that shows that the AEF contralateral to the unilateral laryngeal collapse is subject to more negative airway pressures compared with horses free from pathology (Rakesh et al., 2008a). Histological examination has shown that collapsing AEFs have focal inflammation and edema similar to that seen in cases of laryngomalacia in humans (McCluskie et al., 2006). It has not been established if these histological changes are causative or a result of trauma induced by the airflow during collapse (McCluskie et al., 2006). In humans with EILO, there is virtually no knowledge on basic pathophysiology; however, the aerodynamic principles described in horses are one of the main causal theories currently under investigation (Halvorsen et al., 2017). The larynx is protected from inspiratory collapse by a rigid cartilage skeleton, particularly the cricoid cartilage, and by muscular actions that provide support for the glottis and supraglottic structures. Reidenbach (1998) suggests that a possible reason for the inward collapse of the AEF may be insufficient anchorage to the cartilage skeleton of the larynx. A flaccid, edematous, swollen and/or superfluous mucosa of the arytenoids and of the AEF may contribute by disturbing the airflow, thereby inducing change from laminar to turbulent flow at an earlier stage. A retroflex or omega-shaped epiglottis may contribute in a similar way or represent an obstruction by itself (Belmont and Grundfast, 1984). Airway pressure readings in humans with EILO may provide an objective answer to this, as they have done in horses. Other theories include laryngeal hypersensitivity, neurological reflex arc alterations, and psychological stresses; however, currently all theories lack supporting evidence. Given the complex functions of the larynx, it is likely that the pathophysiology of EILO is multifactorial, and that the etiology varies between individuals, as seems to be the case in horses (Tan et al., 2005). It seems likely that an anatomical or physiological anomaly predisposes the AEFs to collapse at the increasing negative intraluminal pressures induced by the greater airflow of exercise. This theory finds some support by findings in equines of significant associations between severity of deviation of the AEF and increasing number of upper airway abnormalities detected (Tan et al., 2005; Strand and Skjerve, 2012). Figure 2 shows the endoscopic view of MDAF and supraglottic EILO.

Treatment of MDAF is in principle similar in horses and in the corresponding scenario of supraglottic collapse in humans; i.e., mild cases in horses are treated with rest and incremental return to training (King et al., 2001). In humans, mild cases are offered information, biofeedback and breathing control techniques. Cases with more severe collapse are offered biofeedback, or surgical removal of excess aryepiglottic tissue in appropriate highly motivated patients (Maat et al., 2007). In equines, surgery is the primary treatment option for moderate to severe cases (King et al., 2001). Transendoscopic laser excision of the aryepiglottic fold in horses is done under standing sedation and local anesthetic, the excessive membranous AEF tissue is either unilaterally or bilaterally removed with laser excision (King et al., 2001). In humans there is no international consensus on surgical techniques, at the authors’ institution the supraglottoplasty is performed under general anesthesia; releasing incisions to the AEF and removal of excessive tissue around the cuneiform cartilages with laser or microlaryngeal scissors (Maat et al., 2011). For both equines and humans, surgical treatment is reported to have a better subjective outcome compared with conservative treatment (King et al., 2001; Maat et al., 2011). Patients and trainers report a faster return to training and competition and a greater improvement in symptoms. In both equines and humans, it should be noted that conservative treatment is more time consuming, and requires greater efforts and compliance (King et al., 2001; Maat et al., 2011). Importantly, the effect of placebo after surgery should not be underestimated, and controlled trials utilizing sham treatment are certainly warranted (Maat et al., 2011).

## Left Laryngeal Neuropathy

In equines and humans’ failure of full abduction of the arytenoid cartilage may occur unilaterally or bilaterally; however, only unilateral failure will be reviewed here, as bilateral failure is uncommon and results in dyspnea at rest and is as such not a dynamic condition which is the focus of this review (Lane et al., 2006b).

The most common equine cause of left sided failure of arytenoid abduction is the condition “recurrent laryngeal neuropathy,” caused by distal neuronal axonopathy of the recurrent laryngeal nerve (Dixon et al., 2001). In more advanced cases, this failure of abduction may be seen during resting laryngoscopy, which makes it a non-dynamic condition. It is important to view the larynx during exercise in “early” cases, as the grade of collapse can improve as well as worsen during exercise, due to variations in the recruitment of muscle fibers with corresponding variations of the clinical course (Dixon et al., 2001; Lane et al., 2006a).

**FIGURE 3:**
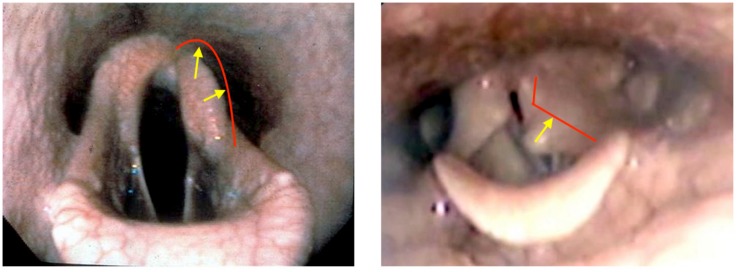
Endoscopic photographic images during exercise of: **(left)** left-sided recurrent laryngeal neuropathy in an equine. **(Right)** Left laryngeal hemiplegia in a human. Red lines denote expected position of arytenoid cartilage in a normal subject. Yellow arrows denote degree of collapse to normal.

In humans, damage to the left recurrent laryngeal nerve during surgery for patent ductus arteriosus, mediastinal, and head and neck surgery is a recognized surgical complication and the leading cause of left sided laryngeal hemiplegia (Røksund et al., 2010; Butskiy et al., 2015; Siu et al., 2016). This condition corresponds with equine “recurrent laryngeal neuropathy” grade 4 (end stage), as this implies complete disruption of motor innervation to the posterior cricoarytenoid (PCA) muscle (Figure 3). In equines, most notably thoroughbreds, this is a severe performance limiting upper airway inspiratory obstruction that requires surgical intervention to restore racing performance (Beard and Waxman, 2007). The surgery most commonly used is the “tie back” with ventriculocordectomy (Beard and Waxman, 2007). This fixates the left arytenoid in an abducted position with removal of one or both vocal folds and laryngeal ventricules. Work with computational fluid dynamics models has shown the optimal degree of abduction is 88% of maximal glottic cross sectional area to allow maximal ventilation without aspiration (Rakesh et al., 2008a). This results in a mild permanent narrowing of the airway, which leads to an increase in the air velocity through this position of the airway, causing a disproportionately greater negative pressure past the arytenoid which pulls the ipsilateral vocal fold into the airway (Rakesh et al., 2008a). Removal of the ipsilateral vocal fold prevents this secondary collapse. Other treatments that have been used include other surgical techniques for permanent fixation or removal of the left arytenoid (Williams et al., 1990), and more recently re-innervation and electrostimulation of the dorsal PCA (Fulton et al., 2003), which have proven successful and returns the function of the arytenoid to normal. Unfortunately, re-innervation takes too long in racing horses to presently be a viable treatment. Furthermore, early detection of denervation before muscle loss is fundamental to return to function in this treatment model (Fulton et al., 2003).

In humans, the aim of treatment is different to that in equines. The priority of the treatment is to decrease the risk of aspiration and to improve vocalization (Butskiy et al., 2015), as opposed to achieving maximal ventilatory capacity without aspiration. Consequently, the aim of treatment is to fix the vocal fold in a relatively adducted position. There are four major approaches to achieve this; injection laryngoplasty, thyroplasty, arytenoid adduction, and reinnervation of the thyroarytenoid muscle (Butskiy et al., 2015; Siu et al., 2016). All four approaches are considered to provide equally good outcomes (Butskiy et al., 2015; Siu et al., 2016). However, these outcomes are necessarily based on the treatment aims, as such, the significant airway obstruction that results from medialization of the vocal fold, especially during increased activity, is not considered. A treatment such as re-innervation of the PCA muscle, that has been successfully applied in equines and restored near normal function, could be a possible future option in humans. Based on what has been learnt from equine research, and that re-innervation has already been used successfully in bilateral vocal fold paralysis in humans, this technique shows future promise for hemiplegia patients with viable PCA muscle (Li et al., 2013).

Other types of malpositioning of the arytenoids that result in airway obstruction include medial luxation of one arytenoid apex under the other (Dart et al., 2005). In equines this is termed Ventromedial Arytenoid Displacement (VMAD) (Dart et al., 2005). This is hypothesized to be due to the transverse arytenoid muscle not being able to support the axial aspect of the arytenoid (Barakzai et al., 2007). One study of VMAD noted post-mortem that the transverse arytenoid ligament was excessively wide. It is clear that further research is needed to determine the pathophysiology of this disorder (Barakzai et al., 2007).

In humans, scissoring of the corniculate cartilages has been anecdotally described, seemingly with a similar visual appearance to VMAD. VMAD results in obstruction of airflow through the most posterior part of the glottic opening. Computational flow dynamics models have shown that the main course of airflow is through the posterior portion of the glottis, above the vocal process (Rakesh et al., 2008a; Frank-Ito et al., 2015). So, although malpositioning of the arytenoids does not visually appear to represent a significant obstruction, it may indeed prove to be, as it obstructs the main channel of airflow. Further research is needed to confirm or refute this in clinical cases. Airway pressure measurements are currently being utilized to evaluate all types and degrees of upper respiratory tract obstruction in equines, including VMAD; hopefully providing information that will aid our understanding of the significance of dynamically malpositioned arytenoids also in humans.

## Vocal Cord Dysfunction in Humans and Equines – Combined Supraglottic and Glottic Collapse in Humans Versus Dynamic Laryngeal Collapse in Equines

**FIGURE 4:**
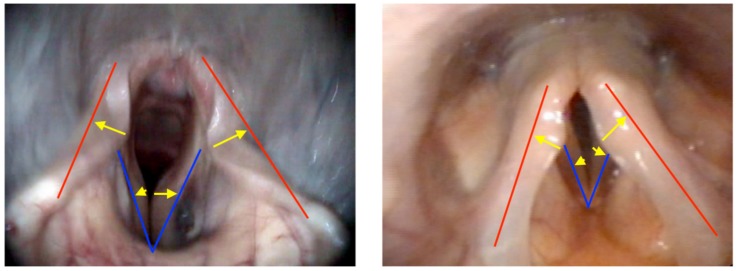
Endoscopic photographic images during exercise of: **(left)** moderate dynamic laryngeal collapse in an equine. **(Right)** Grade 3 combined supraglottic and glottic EILO in a human. Red lines denote expected line of aryepiglottic fold in a normal subject. Blue lines denote expected line of vocal fold in normal subject. Yellow arrows denote degree of collapse to normal.

Vocal cord dysfunction (VCD) is a controversial human diagnosis, and for the purpose of this discussion we will consider it only in response to exercise as a trigger. In equines, vocal fold collapse in absence of collapse of other structures is uncommon but has been described and experimental studies have shown that cricothyroid muscle dysfunction due to superior laryngeal nerve damage is the underlying mechanism (Holcombe et al., 2006). This results in a lack of tension on the vocal folds, which results in a passive drawing into adduction when the ventilatory volume increases. In humans, VCD may occur as the only or the primary obstruction but more commonly occurs secondary to supraglottic (i.e., aryepiglottic fold) collapse (Roksund et al., 2009; Roksund et al., 2017), giving a visual impression similar to what is labeled DLC in equines (Figure 4).

Dynamic laryngeal collapse occurs during poll flexion (flexion of the head relative to the neck) and results in passive collapse of the vocal folds, arytenoid cartilages, and in many cases the AEF (Strand et al., 2009). The underlying mechanism seems related to phenotype since innervation to the larynx and associated muscles is normal (Fjordbakk et al., 2015). The clinical phenotype is an equine with a rostral positioned larynx relative to the mandible accompanied by a narrow intermandibular space (Fjordbakk et al., 2013). This results in the larynx being compressed by the hyoid apparatus during head/neck flexion, preventing full abduction of the arytenoid cartilages during exercise (Fjordbakk et al., 2013).

In both humans and equines, VCD and DLC cause inspiratory obstruction at the glottic level, frequently causing near complete obstruction of the glottis, characterized by a loud stridor (Tan et al., 2005). Glottic obstruction appears to cause human patients the most distress, and cases associated with panic attacks often have a glottic component (Leo and Konakanchi, 1999). A noteworthy possible difference between glottic collapse in equines versus humans, is that the vocal folds are drawn in passively in equines, due to lack of maintenance of arytenoid abduction (Holcombe et al., 2006; Fjordbakk et al., 2008). Contrary, in humans, active contraction of the vocal folds is the most common cause of glottic obstruction (Leo and Konakanchi, 1999). In humans, treatment is aimed at regaining normal control of the vocal folds and preventing inappropriate contraction during inspiration. This can be achieved by biofeedback, speech therapy, inspiratory muscle training (IMT) and in some refractory cases botox (Roksund et al., 2017). In horses, biofeedback and speech therapy are not possible due to the required need to communicate the techniques to the patient. IMT is currently in the initial stages of trialing. The most common treatment in equines has been surgical resection of the vocal folds (Beard and Waxman, 2007). This removes some of the obstructing tissue with tolerable side effects, as domesticated equines do not rely on vocal communication (Beard and Waxman, 2007). However, in cases of DLC it has not proven effective, indicating that the arytenoid cartilage collapse is the main cause of obstruction (Fjordbakk et al., 2008).

## Epiglottic Retroflexion (Retroversion)

**FIGURE 5:**
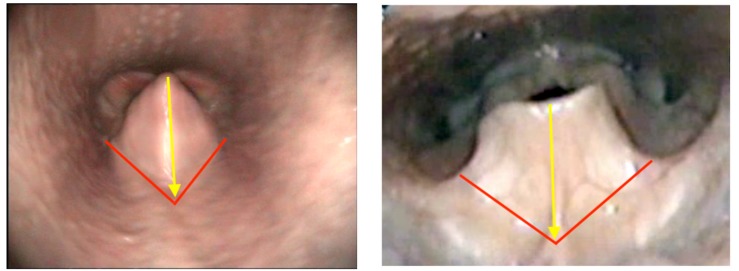
Endoscopic photographic images during exercise of: **(left)** epiglottic retroversion in an equine. **(Right)** Epiglottic retroversion in a human. Red lines denote expected margins of epiglottis in a normal subject. Yellow arrows denote degree of collapse to normal.

Epiglottal retroflexion (ERF) arises when the apex of the epiglottis retroflexes and thereby covers the rima glottis, causing an obstruction of the entrance to the larynx (Figure 5) (Ahern, 2013; Roksund et al., 2017). A collapse of margins of epiglottis can also occur in equines. This condition is rare in humans and uncommon in equines (Tan et al., 2005; Ahern, 2013). In humans, it is often associated with an epiglottis that is omega shaped or with a high resting position and can be seen as a form of supraglottic collapse. The range of retroflexion varies from an epiglottis that fails the anterior rotation normally seen as exercise intensity increases, thereby disrupting airflow, to the epiglottis completely retroflexing into the rima glottis. In equines, the condition has been associated with neuromuscular dysfunction of the hyoid musculature and with damage to the hypoglossal nerve, and consequently hypoepiglotticus muscle paralysis or dysfunction (Ahern, 2013). In a clinical commentary by Ahern (2013), the most common cause of epiglottic retroflexion in equines was trauma to the hypoglossal nerve as a complication of previous upper respiratory tract surgery, other causes included idiopathic and upper respiratory tract infection.

Treatment is based on preventing or limiting retroflexion of the epiglottis into the rima glottis. In humans, the epiglottis is sutured to the tongue base (epiglottopexy). This is not possible in equines due to the conformation of the soft palate in this species. In equines, injection of polytetrafluoroethylene into the epiglottic base, “subepiglottic augmentation,” has been described and was successful in one case (Tulleners and Hamir, 1991). Another described technique is injection of substances to induce friction in the articulation of the epiglottis with the larynx, but this technique has not been critically evaluated in published clinical trials. In both equines and humans, resection of a portion of the apex of the epiglottis can also be performed to reduce the level of obstruction (Ahern, 2013). Currently, prognosis for treatment in both species is unknown, as there are too few cases to compare outcomes (Ahern, 2013).

## Conclusion

Equines and humans are the predominant species that compete at speed and as such are susceptible to airway disorders that are induced at high exercise intensities. As this review demonstrates, dynamic obstructions of the larynx are a common cause of exertional dyspnea and reduced exercise performance in both humans and equines. The underlying anatomy, physiology and the visual appearance of a number of types of dynamic collapse are seemingly comparable in the two species. As such, the equine may provide a good model for human disease, advancing our understanding of pathophysiology and treatment in humans, while allowing equines “early access” to potential future cutting-edge diagnostics and treatment developed in human medicine.

## Author Contributions

ZF-K and TH involved in searching, writing, and compiling the manuscript. ZF-K, TH, OR, J-HH, and ES provided concept of the study and funding. HC, MV, CF, ZF-K, TH, J-HH, ES, and OR involved in reviewing, editing, and writing of the manuscript.

## Conflict of Interest

The authors declare that the research was conducted in the absence of any commercial or financial relationships that could be construed as a potential conflict of interest.
